# Exercise reduces activation of microglia isolated from hippocampus and brain of aged mice

**DOI:** 10.1186/1742-2094-10-114

**Published:** 2013-09-18

**Authors:** Rachel A Kohman, Tushar K Bhattacharya, Elzbieta Wojcik, Justin S Rhodes

**Affiliations:** 1 Department of Psychology, University of Illinois at Urbana-Champaign, Beckman Institute, Urbana, IL 61801-3873, USA; 2 Department of Psychology, University of North Carolina Wilmington, 601 S. College Road, Wilmington, NC 28403-5612, USA

**Keywords:** Exercise, Hippocampus, Aging, Microglia, MHC II, CD86, CD206

## Abstract

**Background:**

Aging is associated with low-grade neuroinflammation that includes basal increases in proinflammatory cytokines and expression of inflammatory markers on microglia. Exercise can reduce neuroinflammation following infection in aged animals, but whether exercise modulates basal changes in microglia activation is unknown. Therefore, we evaluated changes in basal microglia activation in cells isolated from the hippocampus and remaining brain following running-wheel access.

**Methods:**

Adult (4 months) and aged (22 months) male and female BALB/c mice were housed with or without running wheels for 10 weeks. Microglia were isolated from the hippocampus or remaining brain. Flow cytometry was used to determine microglia (CD11b+ and CD45^low^) that co-labeled with CD86, CD206, and MHC II.

**Results:**

Aged mice showed a greater proportion of CD86 and MHC II positive microglia. In aged females, access to a running wheel decreased proportion of CD86+ and MHC II+ microglia in the hippocampus whereas aged males in the running group showed a decrease in the proportion of CD86+ microglia in the brain and an increase in the proportion of MHC II+ microglia in hippocampus and brain.

**Conclusion:**

Overall, these data indicate that running-wheel access modulates microglia activation, but these effects vary by age, sex, and brain region.

## Background

Age-related changes in the brain’s primary immune cell, microglia, have been well documented [1]. Specifically, microglial cells in the aged brain show increased expression of the activation-associated markers major histocompatibility complex II (MHC II), ionized calcium binding adaptor-1 (Iba-1), CD86, ED1 macrophage antigen, CD4, and leukocyte common antigen [1-4]. Additionally, aging is associated with basal increases in proinflammatory cytokine levels, including interleukin-1β (IL-1β), IL-6, and tumor necrosis factor-α (TNF-α) [5-8]. Collectively these findings indicate that microglia in the aged brain are primed towards an inflammatory phenotype that likely contributes to the development of an environment characterized by chronic low-grade neuroinflammation.

The age-related priming of microglia has been linked to a variety of neuropathologies. For instance, the basal changes in microglia activity and proinflammatory cytokine levels are associated with deficits in cognitive function as well as measures of neural plasticity such as hippocampal neurogenesis and long-term potentiation (LTP) [4,9-12]. Additionally, age-related changes in microglia activity have been proposed to contribute to the progression of neurodegenerative diseases, such as Parkinson’s and Alzheimer’s disease, however the precise role of microglia in these conditions is still a matter of debate [13,14]. Collectively, the data indicate that age-related changes in microglial cells, even in the absence of an immune challenge, can impact cognitive function and neural plasticity, and may contribute to disease progression.

In addition to age, there is evidence that suggests an organism’s sex may influence microglia activation. For instance, microglia isolated from neonatal males show increased IL-1β expression following an immune challenge compared to microglia from females [15]. Similarly, Lenz et al. [16] reported that on postnatal day 2 males had increased numbers of microglia and elevated markers associated with activation compared to females. These sex differences appear to persist into old age as prior work has shown aged females show an increase in the total number of microglia in the hippocampus compared to adult females whereas aged and adult males do not differ [17,18]. The existing reports indicate that sex may be an important factor in regulating microglial cells, but additional work is needed to further characterize the differences that exist between males and females and how sex may influence age-related changes in microglia.

One intervention that is known to modulate immune function is aerobic exercise [19-25]. The effects of exercise on immune function are not limited to the periphery, but also occur within the brain. For instance, Nichol et al. [22] reported that exercise significantly reduced the number of CD11b positive cells in the hippocampus of aged transgenic Alzheimer’s mice. Work by Barrientos et al. [26] showed that wheel-running prevented the age-related increase in susceptibility to cognitive deficits following *E*.*coli* infection. Further they reported that microglia isolated from the hippocampus of aged exercising rats showed reduced expression of IL-1β, TNF-α, and IL-6 following endotoxin administration compared to cells isolated from control aged rats [26]. Our laboratory found that voluntary wheel-running decreases hippocampal expression of several immune related genes (for example, MHC I and complement component 4) that were elevated in aged mice [27]. Additionally, we have reported that exercise decreases basal levels of microglia proliferation in aged mice compared to aged control mice and increases the proportion of microglia within the hippocampus that co-label with the neuroprotective factor insulin-like growth factor-1 (IGF-1) [28]. Collectively, this work indicates that exercise may have anti-inflammatory effects within the aged brain, but the extent to which exercise can modulate the basal phenotype of microglial cells is not known.

The current study set out to determine whether voluntary wheel-running alters the activation status of microglia by assessing basal levels of markers associated with microglia activation in both adult and aged mice. Based on prior research, we hypothesized that microglia from aged mice would show increased expression of activation markers compared to adults and that running-wheel access would attenuate this response. In addition, the present report assessed potential sex-related differences in basal microglia activation and whether sex influenced the response to voluntary wheel-running. To test these hypotheses we isolated microglia from the hippocampus and remaining brain regions of adult and aged male and female mice. Flow cytometry was used to distinguish microglial cells from macrophages using antibodies against CD11b and CD45, as microglia are CD11b positive and show low expression of CD45 whereas macrophages are positive for CD11b and show high expression of CD45. Additionally, microglia activation was assessed by examining the proportion of MHC II and CD86 positive microglia. Activation towards the alternative neuroprotective or M2 phenotype was determined by assessing the proportion of microglia positive for the mannose receptor (CD206).

## Methods

### Animals

Subjects used in Experiment 1 were adult male (4 months, *n*=18) and female (4 months, *n*=18) and aged male (21–22 months, *n*=14) and female (21–22 months, *n*=14) BALB/c mice purchased from Charles River Laboratories (Roanoke, IL, USA). A second group of aged female mice (22 months, *n*=14) were purchased from the National Institute of Aging colony (maintained by Charles River Laboratories) and used in Experiment 2. Age of animals listed above reflects age at which microglia were isolated. Aged mice in Experiment 1 were purchased at 7 months old and then aged in the AAALAC-approved facility at the Beckman Institute at the University of Illinois. Mice were given *ad libitum* access to food and water and housed under a 12-h reverse light/dark cycle. Animals were treated in compliance with the *Guide for the Care and Use of Laboratory Animals* and the experiments were conducted in accordance with a protocol approved by the Institutional Animal Care and Use Committee (IACUC) at the University of Illinois at Urbana-Champaign (protocol number 09167 and Animal Welfare Assurance Number A3118-01).

### Experiment 1

Mice were divided by age and sex into their exercise condition of either the running-wheel (access to a running wheel) or control group, for a total of eight treatment groups (adult male control=9, adult female control=9, adult male running wheel *n*=9, adult female running wheel *n*=9, aged male control *n*=7, aged female control *n*=7, aged male running wheel *n*=7, aged female running wheel *n*=7). Mice in the control condition were individually housed in standard polypropylene shoebox cages (29 cm L × 19 cm W × 13 cm H). Mice in the running-wheel condition were individually housed in cages (36 cm L × 20 cm W × 14 cm H) with a 23 cm diameter running wheel (Respironics, Bend, OR, USA). Throughout the 10 weeks wheel rotations were continuously collected in 1 min intervals via magnetic switches interfaced to a computer using the VitalView software (Respironics, Bend, OR, USA). Mice were housed under control or running-wheel housing conditions for a total of 10 weeks. All mice were weighed once a week beginning 1 day prior to their assignment to the running-wheel or control group.

After the 10 weeks of wheel-running or control housing, mice were euthanized by CO_2_ asphyxiation and brains were rapidly removed. The whole hippocampus was dissected from the brain. The hippocampus and the remaining whole brains (that is, everything but the hippocampus, including the cerebellum, cerebral cortex, thalamus, cerebellum, and brain stem) were placed into separate tubes containing cold Dulbecco’s phosphate buffered saline (DPBS) with 0.2% glucose (Fisher Scientific Inc., Pittsburg, PA, USA). Microglial cells were isolated from the hippocampus and brain using discontinuous Percoll density gradient (as described below, 2.4). To ensure a sufficient number of microglia was recovered from the samples we pooled tissue samples from two mice within a given experimental group. When an experimental group had an odd number of animals, tissue from three mice from the same experimental group was pooled. The proportion of microglia (CD11b+ and CD45^low^ cells) that co-labeled with MHC II and CD86 was determined on microglia isolated from the brain samples and co-labeling with MHC II and CD206 from hippocampus samples was determined by flow cytometry.

### Experiment 2

Experiment 1 showed minimal effects of running-wheel access on activation of microglia isolated from the brain of aged females. However, microglia isolated from the hippocampus of aged females showed a decrease in the proportion of MHC II positive microglia. In order to strengthen the conclusion that wheel-running was modulating microglia activation in the hippocampus of aged females we conducted a follow-up experiment that assessed the proportion of hippocampal microglia expressing the activation markers MHC II and CD86. In Experiment 2, 14 aged female BALB/c mice were divided into either the running-wheel (access to a running wheel) or control condition, for a total of two treatment groups (aged female control *n*=7 and aged female running wheel *n*=7). Mice were individually housed under control or running-wheel conditions for 8 weeks. Mice were euthanized by CO_2_ asphyxiation. Tissue samples were pooled from two to three mice within an experimental group and placed into tubes containing cold DPBS with 0.2% glucose. Microglial cells were isolated from the hippocampus and brain using discontinuous Percoll density gradient. The proportion of microglia (CD11b+ and CD45^low^ cells) that co-labeled with MHC II and CD86 was determined on microglia isolated from hippocampal samples by flow cytometry.

### Microglia isolation

Brain and hippocampal samples were homogenized by passing the tissue through a 70 μm nylon mesh cell strainer into a 50 mL conical tube containing DPBS with 0.2% glucose. Samples were spun in a refrigerated centrifuge at 600 × g for 6 min at 10°C. Supernatant was aspirated and cells were resuspended in 70% isotonic Percoll (Fisher Scientific Inc., Pittsburg, PA, USA). The cells suspended in 70% Percoll were then transferred to a 15 mL conical tube and layered with 50%, 35%, and 0% Percoll and then centrifuged at 2,000 × g for 20 min at 10°C with no brake. Microglial cells were collected from the 70% and 50% interface and transferred to a new tube. Cells were washed with DPBS w/ 0.2% glucose and centrifuge at 600 × g for 6 min at 10°C and then resuspended in FACS/Flow buffer (BD biosciences).

### Flow cytometry

**Figure 1:**
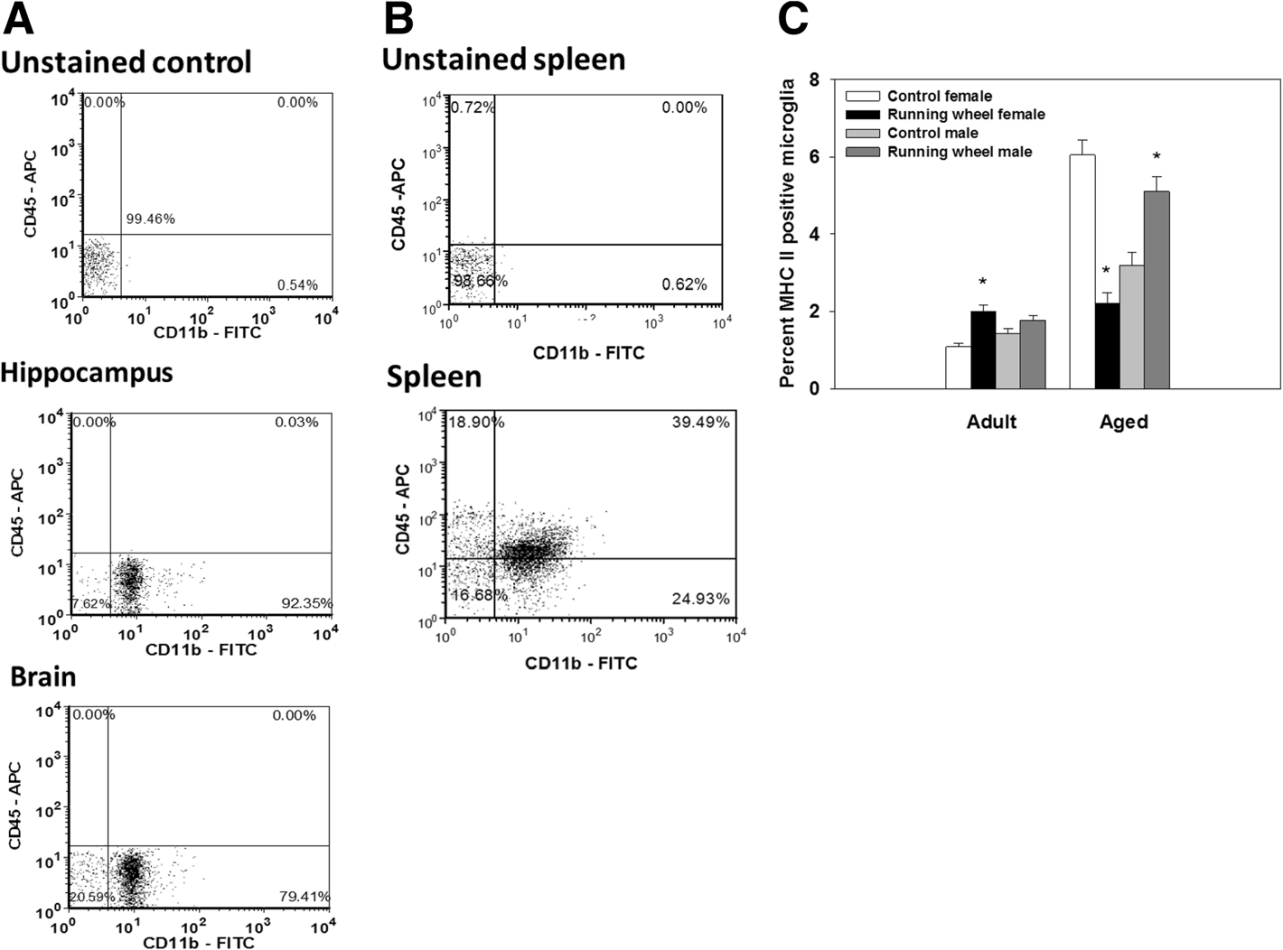
**Proportion of hippocampal microglia positive for MHC II in Experiment 1. (A)** Representative dot-plot of microglial cells (lower right corner) isolated from the hippocampus and brain samples using a Percoll gradient from mice in Experiment 1. **(B)** Representative dot-plot of cells isolated from spleen samples for positive control for CD11b and CD45 labeling. **(C)** Average proportion of microglia isolated from the hippocampus of aged (males *n*=7, females *n*=7) and adult (males *n*=9, females *n*=9) running-wheel and control mice (aged males *n*=7, aged females *n*=7, adult males *n*=9, adult females *n*=9) from Experiment 1 that were positive for MHC II. Wheel-running significantly decreased the proportion of MHC II positive microglia in aged females, but increased the proportion in aged males and adult females. Means ± SEM. * indicates a significant difference from age- and sex-matched control mice.

**Figure 2:**
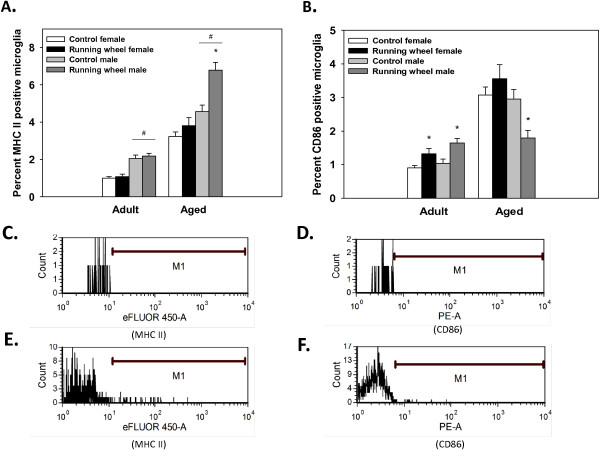
**Proportion of brain microglia positive for MHC II and CD86 in Experiment 1. (A)** Average proportion of microglia isolated from the brains of running-wheel mice and control mice that were positive for MHC II. Aged males (*n*=7), but not aged females (*n*=7) or adult mice (males *n*=9, females *n*=9), showed an increase in the proportion of MHC II positive microglia from running-wheel access. **(B)** Average proportion of microglia isolated from the brains of running-wheel mice and control adult and aged mice that were positive for CD86. Aged males, but not females, showed a significant reduction in the proportion of CD86 positive microglia from running. Bars represent means ± SEM. * indicates a significant difference from age- and sex-matched control mice. # indicates a significant difference from age-matched female mice. **(C)** Histogram of MHC II signal intensity from non-stained control sample; M1 indicates the gate for the MHC II positive cells. **(D)** Histogram of CD86 signal intensity from non-stained control sample; M1 indicates the gate. **(E)** Histogram of representative hippocampal sample labeled for MHC II. **(F)** Histogram of representative hippocampal sample labeled for CD86.

To prevent non-specific binding, all cells were incubated for 5 min with anti-mouse CD16/CD32 (eBiosciences 14016185) to block Fc receptors. A portion of cells from each sample were pooled and used for non-stained control, compensation controls (APC, FITC, PE, eFlour 450), or isotype controls (IgG2a [APC, eBiosciences 551442] and IgG2b [FITC, eBiosciences 11403181]). Hippocampal microglia were then incubated with fluorescent labeled antibodies against CD11b (FITC, eBiosciences 11011285) and CD45 (APC, eBiosciences 14045182) to assess microglia purity and MHC II (eFlour 450, eBiosciences 48532182) and CD206/mannose receptor (PE, AbD Serotec MCA2235PE) for 40 min. Microglia isolated from the brain and hippocampal samples from Experiment 2 were incubated with antibodies against CD11b, CD45, MHC II, and CD86 (PE, eBiosciences 12086282) for 40 min. Following the incubation, cells were washed with flow buffer, fixed using fixation solution (BD biosciences Cytofix/Cytoperm™ Kit), washed with BD wash buffer and then resuspended in flow buffer. Expression of surface antigens was analyzed using a Becton-Dickinson LSR II flow cytometer (Red Oaks, CA, USA). A minimum of 50,000 events were recorded for each sample and analyzed using FCS Express software (De Novo Software, Los Angeles, CA, USA). The average proportion of microglial cells (that is, CD11b+ and CD45^low^) for both experiments from the hippocampus was 86.23% and from the brain 70.95%. Gating for each antibody was determined based on the non-stained control (see Figure 1A). Specifically, the initial gating was based on a dot-plot of the side scatter (SSC) and forward scatter (FSC) that was used to identify monocyte cells (that is, microglia and macrophages) from other cell types as well as distinguish live cells from dead cell. In order to distinguish autofluorescence from a cell positive for a given marker a non-stained control sample was run for each of the fluorochromes and a histogram was constructed to set the gate for each individual marker (see Figure 2C-F). A cell that had a fluorescence intensity that exceeded the autofluorescent gate was considered to be positive for that marker. A second gate was then applied to distinguish microglia from macrophages from all the cells that were monocyte-like based on SSC and FSC in which the cells were plotted on a dot-plot of CD45 and CD11b. Microglia were positive for CD11b and showed extremely low expression of CD45. Our characterization of microglia based on their CD11b and CD45 expression is in agreement with prior reports [29-31]. All of the cells that were identified as microglia were then analyzed for the proportion of CD206, CD86, or MHC II based on the gates set on the non-stained control samples. As a positive control for CD11b and CD45 we labeled cells isolated from spleen samples, these data show cells positive for CD11b and CD45 (see Figure 1B).

### Statistical analysis

Distance ran was analyzed by repeated measures ANOVA with Age, Sex, and Exercise condition (that is, running-wheel or control) as the between-subjects variables and Day as the within-subjects (that is, repeated-measures) variable. The change in body weight and the starting body weight were analyzed by ANOVA. Fisher’s least significant difference post hoc test was used when appropriate. The proportion of microglia isolated from the hippocampus or brain that expressed a particular surface antigen was analyzed by logistic regression. The proportion of microglia expressing CD206, MHC II, or CD86 was calculated by dividing the number of co-labeled microglia by the total number of microglia (CD11b+, CD45^low^) and multiplying by 100 to get a percentage score. For these analyses, the deviance is reported in place of the F statistic. The deviance is equal to −2 times the difference in log likelihood between the parameter-saturated and parameter-reduced model, and is approximately chi-square distributed. Post hoc tests were conducted when significant interactions were found by comparing specific group differences using logistic regression. To correct for multiple comparisons the false discovery rate was employed [32]. An alpha level of *P* < 0.05 was considered statistically significant.

## Results

### Wheel-running distance

#### Experiment 1: Adult and aged males and females

**Figure 3:**
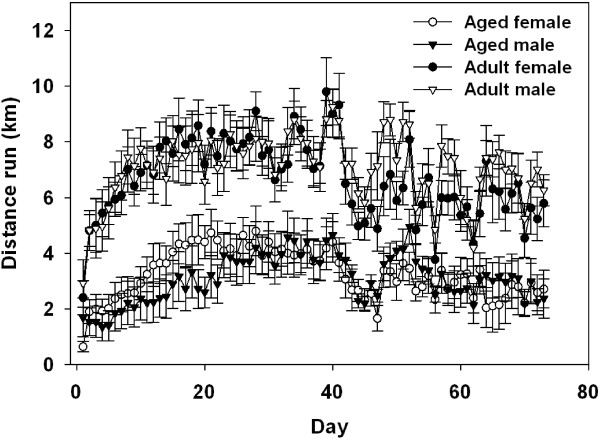
**Average distance (km) run per day by aged and adult male and female mice in Experiment 1 across 10 weeks.** Overall adult mice ran a greater distance than aged mice regardless of the animal’s sex. On average aged males (*n*=7) ran 3.09 km/day, aged females (*n*=7) ran 3.25 km/day, adult males (*n*=9) ran 7.09 km/day, and adult females (*n*=9) ran 6.67 km/day. Means ± standard error of the mean (SEM).

A significant main effect of Age and a significant Age × Day interaction (F (1.28)=37.57; *P* <0.0001; F (72.2016)=3.10; *P* <0.0001, respectively, see Figure 3) showed that on majority of the days adult mice ran a farther distance than aged mice. On average aged males ran 3.09 km/day, aged females ran 3.25 km/day, adult males ran 7.09 km/day, and adult females ran 6.67 km/day, but this varied across days as indicated by a significant main effect of Day (F (72.2016)=42.77; *P* <0.001, see Figure 3).

#### Experiment 2: Aged females

On average aged female mice ran a distance of 5.34 km/day, but this varied across days as indicated by a significant main effect of Day (F (59.295)=5.82; *P* <0.0001, data not shown). The average distance run in Experiment 2 (5.24 km/day) is greater than seen in aged females in Experiment 1 (3.09 km/day). Inspection of the individual data showed this may result from two mice that had an average running distance less than 1.90 km /day in Experiment 1 and two mice in Experiment 2 that averaged above 6.2 km/per overall.

### Body weight

#### Experiment 1: Adult and aged males and females

Analysis of the animal’s body weight prior to being assigned to the running-wheel or control group show a significant main effect of Age (F (1.55)=412.98; *P* <0.001), that showed aged mice weighed more than adults. Additionally there was a main effect of Sex (F (1.55)=110.93; *P* <0.001) that showed males weighed more than females. No pre-existing differences in body weight were present in mice assigned to either the running-wheel or control groups. Analysis of body weight throughout the experiment showed that there were significant main effects of Sex, Age, and Exercise for body weight that showed aged mice weighed more than adults (F (1.55)=256.29; *P* <0.001), males weighed more than females (F (1.55)=184.31; *P* <0.001), and control mice weighed more than running-wheel mice (F (1.55)=6.38; *P* <0.05).

#### Experiment 2: Aged females body weight

A significant main effect of exercise condition showed that at the beginning of the study aged females in the running-wheel group weighed less than the control group (F (1.12)=20.73; *P* <0.005). Analysis of body weight throughout the experiment showed that there was also a significant main effect of Exercise condition (F (1.12)=15.62; *P* <0.001), as aged female mice with access to running wheels (average weight 24.9 grams) weighed less than control mice (average weight 28.3 grams), indicating the initial difference was maintained throughout the experiment.

#### Experiment 1: Expression of MHC II and CD206 on microglia isolated from hippocampal samples of adult and aged males and females

Analysis revealed that aged mice showed a higher proportion of hippocampal microglia that expressed MHC II compared to adult mice, as indicated by a significant main effect of Age (deviance=312.08, *P* <0.0001). There was a significant three-way interaction between Age, Sex and Exercise condition (deviance=59.94, *P* <0.0001, see Figure 1C). For the adult males, there was no difference between the running-wheel mice and control mice, whereas adult females in the running-wheel group showed an increase in the proportion of MHC II positive microglia compared to controls (*P* <0.05). Aged females in the running-wheel group showed a significant reduction in the proportion of MHC II positive microglia relative to controls (*P* <0.05). In contrast, aged males in the running-wheel group showed an increase in the proportion of MHC II positive microglia compared to controls (*P* <0.05; see Figure 1C).

The overall the proportion of CD206 positive microglia were very low, all groups had less than 0.1% of their microglia co-label with CD206, raising the question of the biological relevance of these differences. Though low proportion of CD206 positive microglia were found significant differences were observed as male mice had higher percentages of CD206 positive microglia than females (deviance=12.45, *P* <0.0005, data not shown). Additionally, aged mice had a higher percentage of CD206 positive microglia than adults (deviance=50.77, *P* <0.0001, data not shown). Based on the current data, we cannot rule out the possibility that running-wheel access or age affects the expression of the alternative neuroprotective or M2 phenotype as the possibility exist that other markers (for example, arginase-1 or Ym1) may be better suited to assess phenotypic changes.

#### Experiment 2: Expression of MHC II and CD86 on microglia isolated from hippocampal samples of aged females

**Figure 4:**
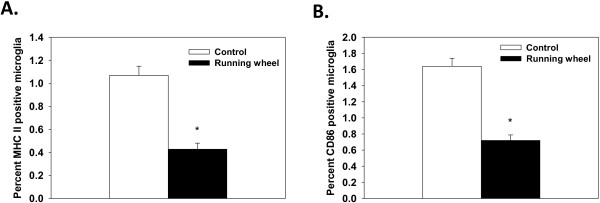
**Proportion of hippocampal microglia positive for MHC II and CD86**, **Experiment 2. (A)** Percentage of hippocampal microglia isolated from running-wheel (*n*=7) and control (*n*=7) aged females from Experiment 2 that were positive for MHC II. Wheel-running significantly decreased the proportion of MHC II positive microglia in the hippocampus. **(B)** Percentage of hippocampal microglia isolated from running-wheel and control aged females that were positive for CD86. The proportion of CD86 positive microglia was significantly reduced by running-wheel access. Means ± SEM. * indicates a significant difference from control mice.

In agreement with Experiment 1, aged females in the running-wheel group showed a significant decrease in the percentage of hippocampal microglia that expressed MHC II compared to control mice (deviance=45.06, *P* <0.0001, see Figure 4A). However, the aged females in Experiment 2 had a lower overall proportion compared to aged females in Experiment 1. While we cannot completely explain this difference, one possibility is variation is the time the animals were housed in colony at UIUC as mice the in first experiment were housed from 7 months to 22 months whereas mice in Experiment 2 were only housed in the colony for a few weeks. Alternatively, the difference may be the result of difference in the distance ran on the running wheels as the aged female mice in experiment 2 ran a farther distance than those in experiment 2. Even with these proportional differences both experiment showed reductions in the percentages of MHC II positive cells following access to a running wheel. Additionally, running-wheel mice had a significantly lower proportion of microglia in the hippocampus that expressed CD86 relative to control mice (deviance=58.88, *P* <0.001, see Figure 4B).

### Experiment 1: Expression of MHC II and CD86 on microglia from microglia isolated from brain samples of adult and aged males and females

There were significant main effects of Sex, Age, and Exercise for the proportion of microglia that expressed MHC II. Males had a greater proportion of MHC II positive microglia relative to females (deviance=86.41, *P* <0.0001; see Figure 2A). Aged mice had a higher percentage than adults (deviance=382.2, *P* <0.0001). Lastly, running-wheel mice had a higher proportion of MHC II positive microglia than control mice (deviance=41.1, *P* <0.0001). Though no significant interaction was found, inspection of Figure 2A shows that aged males in the running-wheel group have an increase in the proportion of MHC II positive microglia compared to aged male controls (*P* <0.05), whereas for adult and aged females and adult males there was no difference between the running-wheel mice and control mice.

There were significant main effects of Age and Exercise for the proportion of microglia that express CD86 within the brain. Aged mice showed a higher proportion of CD86 positive microglia compared to adults (deviance=157.19, *P* <0.0001). Additionally, there was a significant three-way interaction between Age, Sex, and Exercise condition (deviance=6.23, *P* <0.05). As depicted in Figure 2B, aged males in the running-wheel group showed a significant decreased in the proportion of CD86 positive microglia compared to controls (*P* <0.05), whereas in aged females there was no difference between running-wheel mice and control mice. For adult mice in the running-wheel group, there were modest increases in the percentage of CD86 positive microglia compared to control mice (*P* <0.05; see Figure 2B).

## Discussion

The potential immunomodulatory effects of exercise within the brain are of particular interest in the context of aging as normal aging primes microglia towards the classic inflammatory phenotype [1,2,4]. In agreement, our data show that aged mice have a greater proportion of microglia that express MHC II and CD86 in both the hippocampus and the remaining brain relative to adult mice. Additionally, the present study extends the existing literature by demonstrating that voluntary wheel-running, particularly in aged animals, alters basal microglia activation as evidenced by changes in the expression of MHC II and CD86. However, the modulatory effects of running-wheel access on microglia activity are not only dependent on the animal’s age, but also on the animal’s sex and the area of the brain the cells were isolated from.

Robust differences related to the animal’s sex were observed. Even in adults, males had higher basal proportion of microglia positive for MHC II within the brain compared to adult females. While both aged males and females had significantly higher proportions of MHC II and CD86 positive microglia compared to adults, the proportion of MHC II positive microglia was higher in the brain of aged males compared to aged females potentially indicating stronger microglia activation in males. These findings add to prior reports that show sex differences in microglia number and function. For instance, Lenz et al. [16] report that there is a greater number of microglia and more activated cells in the preoptic area of males compared to females on postnatal day 2. Similarly, aged females show an increase in the total number of microglia in the hippocampus compared to adult females, whereas aged males did not show a significant increase in hippocampal microglial cells [17,18]. In terms of function, a recent paper has found that microglia isolated from female neonatal or adult rats show reduced expression of IL-1β following endotoxin exposure compared to cells from males, the authors attribute these sex differences to differences in estrogen levels, [15]. The current findings substantiate the existence of differential microglia activation in males and females.

Alterations in microglia activity in response to running-wheel access interacted with the animal’s age and sex. For adults, wheel-running had minimal effects on the proportion of microglia expressing CD86 and MHC II, though in a few instances increases were observed. The limited effect on microglia from adult mice is expected as majority of these cells are going to be in the resting non-activated state and therefore may be less responsive to external events as compared to the primed microglia in the aged mice. For aged females, running-wheel access reduced the proportion of MHC II and CD86 positive microglia in the hippocampus, indicating a reduction in microglia activation. CD86 is a co-stimulatory molecule known to be expressed on antigen-presenting cells and to be important in the induction of T cell proliferation and activation, potentially indicating running-wheel access is diminishing the likelihood of interactions between microglia and infiltrating T cells within the brain [33]. Similarly, aged males showed a reduction in the proportion of CD86 positive microglia isolated from the brain. However, the proportion of MHC II positive microglia was increased in both the hippocampus and brain of aged males. MHC II is commonly used as a marker of microglia priming in the aged brain. Alone MHC II only indicates that microglial cells are activated, but does not differentiate the phenotype the cells have acquired, as MHC II is expressed when microglia express a range of phenotypes (for example, the classic inflammatory M1 or alternative neuroprotective M2 phenotype) [34]. To distinguish the phenotype, MHC II is often measured in conjunction with other surface markers or cytokine expression. The present findings indicate that while running-wheel access is increasing microglia activation in aged males, it is likely altering the form of this activation as evidenced by the changes in the proportion of CD86 positive microglia. In some respects increased activation in microglia is not surprising, as running-wheel access is known to increase neural activity as measured by immediate early gene expression [20]. Additionally, exercise can be viewed as a form of stress on the brain, as exercise induces a host of changes including cell death, neurogenesis, angiogenesis, and changes within the extracellular environment that may initiate microglia activation possibly to assist with adapting to the exercise-induced changes within the brain [20,35-37].

The present data also indicate that voluntary wheel-running had region specific effects on microglia activation. Prior reports have noted regional differences in microglia density as well as the phenotype they express [34,38,39]. Our data showed that while running-wheel access reduced the proportion of CD86 positive microglia in the hippocampus of aged females, no difference was observed in microglia isolated from the brain. It is currently unclear whether the regional differences observed reflect inherent differences in the microglial cells or their reaction to variations in the microenvironment in these different brain areas. Microglia that reside within the hippocampus appear to be unique in some respects relative to microglia in other brain regions. For example, the hippocampus has a higher density of microglia than other brain regions [38]. Additionally, in culture, microglia isolated from the hippocampus show an increased expression of TNF-α, CD4 and Fc gamma receptor II compared to microglia isolated from the cortex or cerebellum [40]. In addition, the hippocampus is particularly responsive to exercise; as neural activation as measured by c-Fos induction is positively correlated with the distance an animal runs [20,41]. Potentially, microglia residing in the hippocampus may respond to the exercise-induced activation of cells in their surrounding environment. Similarly, exposure to 2 weeks of restraint stress was reported to in increase the density of Iba-1 staining in only nine of 15 brain regions assessed indicating regional differences in the response to an external stressor [42]. Another possibility is that the lack of an effect of running-wheel access on microglia isolated from the remaining brain may result from combining cells from a variety of brain areas potentially masking changes in microglia activity that may be occurring in select regions and only allowing for changes that occur more globally throughout the brain to be detected. Currently it is unclear how these regional variations in microglia numbers and activity influence neural function, but further investigation into the region specific characteristics of microglia will help elucidate the specific roles microglia may play in their area of residence.

In summary, the present work indicates that voluntary wheel-running modulates microglia activity, particularly in aged animals, and provides novel evidence that an animal’s sex is an important variable in mediating the response to exercise. While both aged males and females showed evidence of altered microglia activation the nature of these changes were different in males and females and varied by the region the microglial cells were isolated from. The current data in conjunction with prior reports [26-28] indicates that exercise may be an effective intervention to prevent or reverse the age-related changes in immune activity within the brain.

## Abbreviations

DPBS: Dulbecco’s phosphate buffered saline; Iba-1: Ionized calcium binding adaptor-1; IGF-1: Insulin-like growth factor-1; IL-1β: Interleukin-1β; IL-6: Interleukin-6; LTP: Long-term potentiation; MCH II: Major histocompatibility complex II; TNF-α: Tumor necrosis factor-α; FSC: Forward side scatter; SSC: Side scatter.

## Competing interests

All authors declare that there are no competing interests.

## Authors’ contributions

RK conceived of the study and design, participated in microglia isolation, conducted flow cytometery, and drafted the manuscript. TB participated in microglia isolation, assisted with conducting flow cytometery, and edited the manuscript. EW collected weight data and participated in microglia isolation JR assisted with the experimental design, analyzed the data, and edited the manuscript. All authors read and approved the final manuscript.
